# Implications for domain fusion protein-protein interactions based on structural information

**DOI:** 10.1186/1471-2105-5-161

**Published:** 2004-10-26

**Authors:** Jer-Ming Chia, Prasanna R Kolatkar

**Affiliations:** 1 The Genome Institute of Singapore, Singapore

## Abstract

**Background:**

Several *in silico *methods exist that were developed to predict protein interactions from the copious amount of genomic and proteomic data. One of these methods is Domain Fusion, which has proven to be effective in predicting functional links between proteins.

**Results:**

Analyzing the structures of multi-domain single-chain peptides, we found that domain pairs located less than 30 residues apart on a chain are almost certain to share a physical interface. The majority of these interactions are also conserved across separate chains. We make use of this observation to improve domain fusion based protein interaction predictions, and demonstrate this by implementing it on a set of *Saccharomyces cerevisiae *proteins.

**Conclusion:**

We show that existing structural data supports the domain fusion hypothesis. Empirical information from structural data also enables us to refine and assess domain fusion based protein interaction predictions. These interactions can then be integrated with downstream biochemical and genetic assays to generate more reliable protein interaction data sets.

## Background

Networks of interacting molecules drive every process in biological cells. Proteins dominate these networks, some of which involve transient interactions such as signal transduction cascades and ligand-receptor interactions, while others form more permanent molecular machineries such as ribosomes and polymerases. Unraveling these networks and interactions will not only help us better understand complex cellular processes, but also enable us to make inferences about the function of individual proteins through 'guilt-by-association' [1].

Over the last few years, high-throughput interaction detection assays have been introduced and refined to complement the traditional genetic and biochemical techniques. High-throughput mass spectrometry protein complex identification (reviewed by Pandey and Mann [2]), and yeast two-hybrid systems [3] are examples of these. The success of these techniques is well illustrated in the budding yeast *Saccharomyces cerevisiae, *in which networks of its interacting proteome where constructed using genome-wide screens. [4-7]

The wealth of genomic and protein sequences, the increase of 3D structures of protein complexes, together with the deluge of microarray expression data, has provided researchers with an overwhelming body of information that can be used to infer both functional as well as interaction linkages. Clearly, bioinformatics and computational biology are necessary tools for delineating this information. In response to the data explosion, several *in silico *methods have recently been developed to predict associations from these data.

Phylogenetic profiles focus on the co-occurrences of genes across several organisms. By studying the pattern of evolutionary conservation between sets of genes in different organisms (phylogenetic distribution), it has been shown that these phylogenetic profiles can be successfully used to infer both localization as well as functional association between proteins [8-10]. Protein domains that are found fused together within a protein are frequently involved in the same process, and in many examples proven to be physically interacting. This phenomenon is the basis for the domain fusion analysis, which can be used to predict protein interactions in cases where the fused domain pair is found independently across separate protein chains [11,12].

Structural data has also been mined and analyzed for residue patterns within interfaces between pairs of interacting proteins. These are then used to train learning models for *ab initio *categorization and prediction of protein interactions [13,14]. Jansen and co-workers [15] illustrated how expression profiles from mRNA expression data could be harnessed and used as an effective source for the prediction of protein interactions.

A number of groups have compared and reported on the protein interaction datasets that are emerging from the various genome-scale biochemical, genetic and *in silico *experiments [16-18]. All of them drew a similar conclusion; high-throughput methods produce little overlapping results, and taken singularly, each technique has a high error rate (false positive and false negative). Each of these methods has their own specific strength and weakness, and covers a separate subset of interactions. Integrating the various result sets together, allows one to piece together a map of the interacting proteome that is more reliable with higher accuracy, and more informative with higher coverage.

The study by von Mering and co-workers [17] showed that *in silico *methods have higher coverage and higher accuracy than the majority of biochemical/genetics methods, second only to high-throughput mass spectrometry. The use of sensible strategies and filters has allowed *in silico *analyses to provide better performance. On top of that, these methods are less biased towards abundant proteins. *In silico *analyses are indispensable, and further improvements of these methods to make them more accurate will provide a cleaner set of data for downstream biochemical/genetic studies.

In this study, we make use of an empirical observation that domain pairs, which lie in close proximity on a protein chain tend to interact, to refine the domain fusion analysis. This way, we aim to improve the accuracy of the domain fusion analysis.

### Domain fusion

The basis for domain fusion (or gene fusion) is the observation that certain proteins (termed the Rosetta stones) in a given species are found to consist of a fusion between two separate proteins in another species. Through fusion, the entropy of dissociation between the two proteins is reduced, and it is hypothesized that in all likelihood, these two separate proteins share a functional association, if not a physical interaction [11,12].

Domains have been described as the primary building blocks of proteins [19], recombining in various permutations, resulting in proteins of completely different functions [20]. In our implementation of the domain fusion analysis, we chose the representation of proteins being composed of domains, separated by linkers on a peptide chain.

In this paper, we make use of existing structural data to support the domain fusion hypothesis. We interrogated known 3D structures for evidence of inter-domain physical interactions on the same chain. We investigated and concluded that there was an association between the distances at which domains are spaced apart on the chain, and the propensity for a domain-pair to interact.

We also show that domain pairs, located in close proximity on a protein chain, are likely to interact even when found residing on different chains, hence proving that the domain fusion hypothesis is valid.

Finally, we demonstrate that peptide chains with closely spaced domains are likely to make better Rosetta stones, and we make use of this observation to improve domain fusion based protein interaction predictions.

## Results

### Supporting the domain fusion hypothesis

The available structural data indicate that intra-chain domain pairs, which lie in close proximity on a peptide chain, tend to physically interact with one another. The mean distances of interacting intra-chain domain pairs are smaller than ones which do not interact; interacting pairs are on the average 50 residues apart, while non-interacting pairs have a mean distance of 166 residues between them.

In order to verify the correlation between distance and interaction, we made use of contingency tables and the chi-squared test statistic. For a set of inter-domain distances ranging from 5 residues to 200 residues, we constructed 2 × 2 contingency tables that classified domain pairs according to two criterions; 1) whether or not they are separated by a distance no greater than a threshold, and 2) whether or not the domain pair is interacting. The chi-squared value of each table was used as a statistic to test the null hypothesis (*H*_0_): Domains pairs separated by no greater than a predefined distance and their tendency to interact were independent. The p-values indicate the probability of having the chi-squared test statistic as extreme as, or larger than observed when *H*_0 _is true.

**Figure 1:**
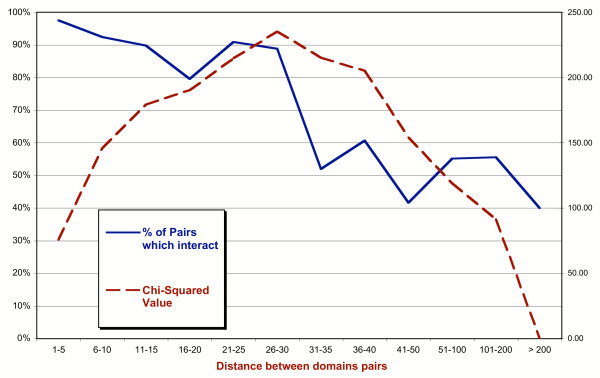
**Distance between domain pairs on a protein chain and the likelihood that they interact **The solid line indicates the percentage of domain pairs, within a distance range apart, which are in contact. The broken line shows the distribution of chi-squared values corresponding to constructed 2 × 2 contingency tables that classified domain pairs according to 2 criterions; 1) whether or not they are separated by a distance no greater than the upper limit of each range, and 2) whether or not the domain pair is interacting. The percentage of interacting domain pairs drop noticeably after 30 residues, and the chi-squared value is also maximum at this threshold.

We found that the contingency table for domain pairs spaced up to 30 residues apart had the highest chi-squared value, with a statistically significant p-value of less than 0.001, allowing us to confidently reject *H*_0_. This trend is noticeable in the chart illustrating the proportion of interacting pairs across various inter-pair distances (figure 1). Domain pairs located less than 30 residues apart are almost certainly (90%) to be in contact with each other, whereas only half (51%) of domain pairs with more than 30 residues separation were categorized as physically interacting. The chi-squared value is also overlaid on the chart in a dotted line, representing the test statistic from each corresponding contingency table.

In order to validate the domain fusion hypothesis, we not only need to show that domain pairs on the same chain tend to interact with each other, but importantly, this same domain pair will tend to be in contact if they are located independently across separate chains of a polypeptide complex. From our data, we noticed that 71% of domain pairs, which lie within 30 residues of each other on the same chain, could be found physically interacting across separate chains of a complex. In contrast only 38% of domain pairs lying greater than 30 residues apart are seen to be in contact within a multi-chain complex. Once again, putting this into a contingency table and evaluating the chi-squared statistic we reject the null hypothesis (p-value <= 0.001). In other words, there is a correlation between domain-pairs spaced less than 30 residues apart on a single peptide chain and their tendency to interact across separate chains of a polypeptide complex.

### 30 residues criteria applied to Swiss-Prot proteins

We wanted to verify that the 30 residues criteria could be used as a measure to filter and improve predictions made using the domain fusion methodology. A set of proteins for the budding yeast *S. cerevisiae *was downloaded from Swiss-Prot, and domain fusion based protein interactions were predicted as described in the Methods section. After filtering for promiscuous domains, a total of 9279 protein interactions remained, of which 28% or 2629 were supported by a Rosetta stone with no more than 30 residues between the fused domains.

The functional category assigned to each protein in an interacting pair was used to gauge the plausibility of the interaction; if two different proteins were found physically interacting, one would expect the two proteins to have overlapping functional categories. 62% of the interacting protein pairs, supported by a 30 residue Rosetta stone, have both partners belonging to the same functional category. The same proportion for interacting pairs not supported by a 30 residue Rosetta stone is 48%. This 14% difference is significant with a p-value of less than 0.001, using a two-sample t-test.

## Discussion

*In silico *methods for predicting protein interactions are not only able to match the accuracy of the other genetic, biochemical and biophysical techniques, but also have the added advantage of providing higher coverage [17]. Among the *in silico *methods, domain fusion is an attractive technique because it enables a functional link to be drawn between two proteins based solely on their primary sequence. Still, large-scale sets of high-throughput protein interaction data available today are spurious, more than half of them proving to be false positives [17], the challenge remains to improve the quality of high throughput protein interaction data sets.

Protein interactions can be classified as either permanent or transient interactions. The data from this study were taken from the PDB, where most of the submitted structures are results from x-ray crystallography experiments. Consequently, we believe that the vast majority of our deduced domain and protein interactions are physical, permanent interactions.

Our study of multi-domain, single and multiple-chain protein structures in the PDB gave us two results. First of all, it supports the domain fusion hypothesis suggested by Marcotte and Enright. Secondly, it allows us to conclude that single chain peptides with closely spaced domain pairs make better Rosetta stones, and hence better predictors of protein interactions.

Evident from the set of PDB structures we studied, is a correlation between the distance separating a pair of domains on a protein chain, and their tendency to physically interact with one another. As described by Marcotte and co-workers when they constructed the domain fusion hypothesis for evolution of protein interactions, affinity between interacting pairs of domains may be enhanced when the domains are fused together on the same chain [11]. Consequently, close proximity of the interacting pair on the same chain increases the effective local concentration of the two domains, facilitating the interaction. The biochemical advantage for such an arrangement would explain the tendency for interacting domains to be found close together on a protein chain. Our observation that domain pairs located less than 30 residues apart are almost certainly to share an interface clearly supports this idea.

Previously, Park and co-workers [21]had observed this figure in an unrelated report. In this study, we adopted a different concept of a protein domain – PFAM categories which are essentially sequence-based annotations. Analyzing a substantial set of structural data from the PDB, we also derive at this similar threshold of 30 residues, and show it to be statistically significant.

### Conservation of domain interactions across multi-chain structures

The data from multi-chain PDB structures provide additional support to the domain fusion hypothesis, by showing that most of the intra-chain domain interactions are similarly represented across separate chains of a complex. This provides additional mechanistic evidence that the interaction between the two domains is most probably functional and conserved.

To our knowledge, this is the first time structural data has been used to support the domain fusion hypothesis.

### Functional classification of non-interacting domains in close proximity

We tried to uncover a pattern within the set of closely spaced, yet non-interacting domain pairs. We wanted to detect if there was an over-representation of domains from a specific molecular functional category in this non-interacting list. This list is displayed in Table 1. From the Gene Ontology categories of the domains, it is obvious that a good proportion of domains on the list are involved in DNA/RNA processing activities, as well as catalytic functions, but we didn't observe any statistically significant differences when comparing this non-interacting set with the sets of domain pairs which interact. This could be due to the small number of non-interacting domains in close proximity.

Furthermore, since the interactions we can detect from structural data are more likely to be permanent interactions, it is possible that the reason no physical contact is witnessed between these proximal domains in structural data is because the domains form transient interactions that are not captured in the x-ray crystallography data.

### Hot loops and interactions

We also looked for a relation between protein disorder and interacting domain pairs. We wanted to see if protein domain pairs which interact on the same chain, tend to be linked by a disordered region. To this effect, we used DisEMBL[22]to do the disorder analysis. However, we were unable to infer any relationship between disorder and interacting domains.

### Use of 30 residue criteria to refine domain fusion predictions

Our results from predicting interactions among *S. cerevisiae *proteins indicate that Rosetta stones with domains separated by less than 30 residues do indeed make better domain interaction (and hence protein interaction) predictors.

The set of protein interactions inferred from these Rosetta stones are enriched with more reliable interactions, as judged by using similar function as a criteria. The total number of interactions is reduced to nearly a quarter when employing this method. This allows us to conclude that the number of false positives is reduced, increasing the accuracy of the prediction. Without needing to employ a hard filter, protein interactions predicted using the domain fusion methodology may be ranked according to the quality of the Rosetta stones each interaction is inferred from, allowing one to identify a much smaller subset of more reliable interactions, and use them for downstream analyses.

## Conclusions

We have successfully demonstrated the use of current structural data as a resource for refining current protein interaction predictions, in particular domain fusion predictions. Our data strongly suggests that domain pairs separated by less than 30 residues on a peptide chain are almost certainly to physically interact, and this criterion is useful in accessing protein interactions predicted from Rosetta stone proteins.

Going forward, the availability of a large number of structures through structural genomics programs will facilitate a larger sampling of the domain structure space. New patterns may emerge as use of this data becomes available, allowing better predictions to be made.

## Methods

### Intra-chain domain interactions

We used domain models from the Protein Family database (PFAM) [23] which were mapped onto structures from the Protein Databank (PDB) [24]. The PFAM to PDB mappings were obtained from PFAM data files, and we only considered PFAM entries that were tagged with the type 'Domain'. There are a total of 4169 peptide chains in the PDB that are annotated with more than one PFAM domain, comprising a total of 504 unique PFAM domains present within the data set. In order to obtain a non-redundant representation of these peptide chains, we took clusters of them based on 50% sequence identity, and selected one representative from each cluster. This left us with a set of 565 3D structures of multi-domain peptide chains, comprising a total of 996 distinct domain pairs, of which 478 are unique pairs.

We used the coordinates within the PDB data files to calculate the distances between domains, and to determine if they are interacting. Two domains are judged to be interacting if they share at least five contacting residue pairs, where contacting residues are residue pairs with less than 6Å between their respective alpha-carbon atoms.

### Multi-chain interactions

Using a similar approach to the above, we obtained a set of multi-chain PDB structures in which the previously determined domain-domain interactions can be observed across separate peptide chains within a complex. The ASALIST from the PQS server [25]was used to sift out the biologically significant contacts from the crystal packed structures. Of the 379 domain pairs above, 305 were found on separate chains of a complex, and these were used for the analysis.

### GO functional annotation

PFAM domains were categorized into Gene Ontology (GO) molecular functions and cellular processes using the PFAM2GO data provided by the GO consortium [26].

### *Saccharomyces cerevisiae *protein interactions prediction

In order to assess how distance between domains could be used to improve the domain fusion based protein interaction predictions, we predicted interactions between 6918 proteins from the organism *Saccharomyces cerevisiae *found within the Swiss-Prot database, and gauged the quality of the interactions by looking at the function of each interacting protein.

The steps taken to predict protein interactions based on domain fusion are as follows. Swiss-Prot (release 42.9) and Trembl (release 25.9) [27] protein datasets were first searched for multi-domain proteins, by relying on their PFAM annotations. As above, only PFAM domains of type 'Domain' where considered. These multi-domain proteins were then catalogued as Rosetta Stones. Pairwise domain interactions were inferred by cataloging each distinct domain pair found on every Rosetta Stone protein, together with the number of residues separating the pair. As described by Marcotte and co-workers [11], domain interactions involving the 5% most promiscuous domains were discarded, removing the majority of false positives.

This domain interaction set was then used to predict pairwise protein interactions between the *S. cerevisiae *proteins, by looking at the complement of PFAM domains between each and every pair of proteins, and seeing if there were any Rosetta stone determined domain interactions between the domains of each protein. The protein interactions were sorted into two groups; one group inferred from domain interactions supported by the existence of a Rosetta stone protein with no more than 30 residues between the domain pair, and the other group with no support from a 30 residue Rosetta stone.

To validate these protein interactions, we mapped the proteins to the MIPS comprehensive yeast genome database [28], and looked for interacting protein partners that share the same MIPS functional category. Interactions between pairs that share the same function are more likely to be true.

All the data was stored in a relational database schema implemented in MySQL, with a set of perl modules written for data transaction and manipulation. The Bioperl bioinformatics tool kit [29] was used to parse Swiss-Prot, Pfam and PDB data, as well as to extract coordinates of each atom from each PDB structure.

## Authors' contributions

JMC participated in the design of the study, performed the data and statistical analysis, as well as drafted the manuscript. PRK conceived of the study, participated in its design and coordination, and edited the final draft of the manuscript. Both authors read and approved the manuscript.

## Figures and Tables

**Table 1:** List of domain pairs separated by less than 30 residues but are not interacting

**Domain 1**	**Molecular Function**	**Domain 2**	**Molecular Function**	**No. of Contacts**
2-Hacid_DH	oxidoreductase activity	2-Hacid_DH_C	oxidoreductase activity	4
CH		CH		0
Cytochrome_CIII		Cytochrome_CIII		0
dsrm	double-stranded RNA binding	dsrm	double-stranded RNA binding	0
EGF		EGF		0
eRF1_l		eRFl_2		0
FKBP		FKBP		1
fnl		fnl		0
fnl		fn2		2
fn2		fn2		0
GlutR_NAD_bind	glutamyl-tRNA reductase activity	GlutR_dimer	glutamyl-tRNA reductase activity	1
HTH_9	molybdate ion transporter activity	TOBE		0
kazal		kazal		0
MHC_II_beta	immune response	ig		2
myb_DNA-binding	DNA binding	myb_DNA-binding	DNA binding	0
Peptidase_M10	proteolysis and peptidolysis	fn2		2
Phe_tRNA-synt_N	phenylalanine-tRNA ligase activity	tRNA-synt_2d	phenylalanine-tRNA ligase activity;	0
resolvase	recombinase activity ;DNA recombination	HTH_7	recombinase activity;DNA recombination	1
RHD	regulation of transcription, DNA-dependent	TIG		0
Ribosomal_L9_N	structural constituent of ribosome	Ribosomal_L9_C	structural constituent of ribosome	0
RNase_PH_C	RNA binding;RNA processing	KH	nucleic acid binding	0
Rotamase	isomerase activity	Rotamase rrm	isomerase activity	0
rrm		rrm		4
rve	DNA binding ;DNA recombination	integrase	integrase activity	0
SH2	intracellular signaling cascade	SH3		0
sushi		sushi		4
TPP_enzymes_N		TPP_enzymes		2
WW		WW		0
zf-Sec23_Sec24		Sec23_trunk		0
